# Acute onset of spinal dural arteriovenous fistula and rapid progression to complete paraplegia: a case report and review of the literature

**DOI:** 10.1186/s13256-025-05454-1

**Published:** 2025-08-06

**Authors:** Xinling Su, Liping Huang, Gang Wang, Ming Zhou, Suhang Xie, Yangxiaoxue Liu

**Affiliations:** Department of Rehabilitation Medicine, The First Medical Center of Chinese, PLA General Hospital, No. 28 Fuxing Road, Haidian District, Beijing, China

**Keywords:** Spinal dural arteriovenous fistula, Acute onset, Rapid progression, Endovascular embolization, Case report

## Abstract

**Background:**

Spinal dural arteriovenous fistula usually has an insidious clinical course and is easily misdiagnosed. In some cases, acute exacerbation occurs following certain triggers, such as corticosteroid use and lumbar puncture. Here, we introduce a rare case of spinal dural arteriovenous fistula with acute onset, rapid progression, and no cause.

**Case presentation:**

A 76-year-old Chinese male patient presented with numbness and weakness of the lower limbs that rapidly progressed to complete paralysis within 4 days. Patient was diagnosed with spinal dural arteriovenous fistula after magnetic resonance imaging and spinal vascular angiography, undergoing bilateral internal iliac artery embolization 2 weeks later, and started rehabilitation 40 days later but only received minimal improvement 1 year thereafter.

**Conclusion:**

This case highlights that spinal dural arteriovenous fistulas cannot be excluded in acute-onset or rapidly progressing spinal cord lesions; such patients with severe neurological dysfunction may have a poor prognosis even after prompt surgical treatment.

## Background

Spinal dural arteriovenous fistula (SDAVF) is rare, with an annual incidence of approximately 5–10/1,000,000, and is more common among middle-aged and elderly male individuals [1]. SDAVF is the most common type of spinal vascular malformation, accounting for up to 60–80% of all spinal cord vascular lesions [1–4]. It usually has an insidious onset with progressive aggravation of symptoms that are nonspecific, therefore, the final diagnosis is typically made relatively late after the initiation of diagnostic evaluation when marked neurological deficits develop. Nevertheless, functional outcomes after treatment for SDAVFs are favorable, but the proportion of patients who show improvement varies from 25% to 100% [5–7].

Here, we present a rare case of SDAVF with acute onset, no cause, and rapid progression to paraplegia. The patient was treated surgically but showed only minimal improvement.

## Case presentation

A 76-year-old Chinese male patient was admitted to our rehabilitation medicine department on 7 May 2022 due to progressive numbness and weakness of the lower limbs and urinary and defecation disorders for more than 1 month. The patient had a history of hypertension, type 2 diabetes mellitus, and atrial fibrillation and no history of smoking or alcohol consumption. The patient began experiencing numbness and weakness in both lower limbs without obvious cause while sleeping in bed at approximately 1:00 am on 27 March. Although he was able to walk independently, he experienced leg pain while walking, accompanied by low back pain and dysuresia. The above symptoms gradually worsened, and the patient needed to walk with crutches the next day and could not walk or urinate and defecate 4 days later. Magnetic resonance imaging (MRI) of the cervical, thoracic, and lumbar vertebrae at the local hospital revealed suspicious spinal cord lesions at the T9–12 level, and enhanced MRI of the thoracic vertebrae revealed abnormal enhancement in the middle and lower segments of the thoracic medulla. Lumbar puncture revealed cerebrospinal fluid (CSF) pressure of 100 mmH_2_O, white blood cell count of 10 × 10^6^/L (lymphocytes accounted for 85%), protein concentration of 2.79 g/L, glucose concentration of 4.9 mmol/L, and lactate concentration of 4.47 mmol/L. CSF was negative for pathogens and for immune-associated antibodies including anti-APQ4 antibody, anti-MBP (Myelin Basic Protein) antibody, and anti-MOG (myelin oligodendrocyte glycoprotein) antibody. Patient’s initial diagnosis was considered inflammatory demyelinating myelopathy, and treatment with intravenous immunoglobulin (IG; 37.5 g/d for 5 days) and corticosteroids (dexamethasone 10 mg/d for 5 days) was administered; however, no symptom improvement was observed.

**Fig. 1:**
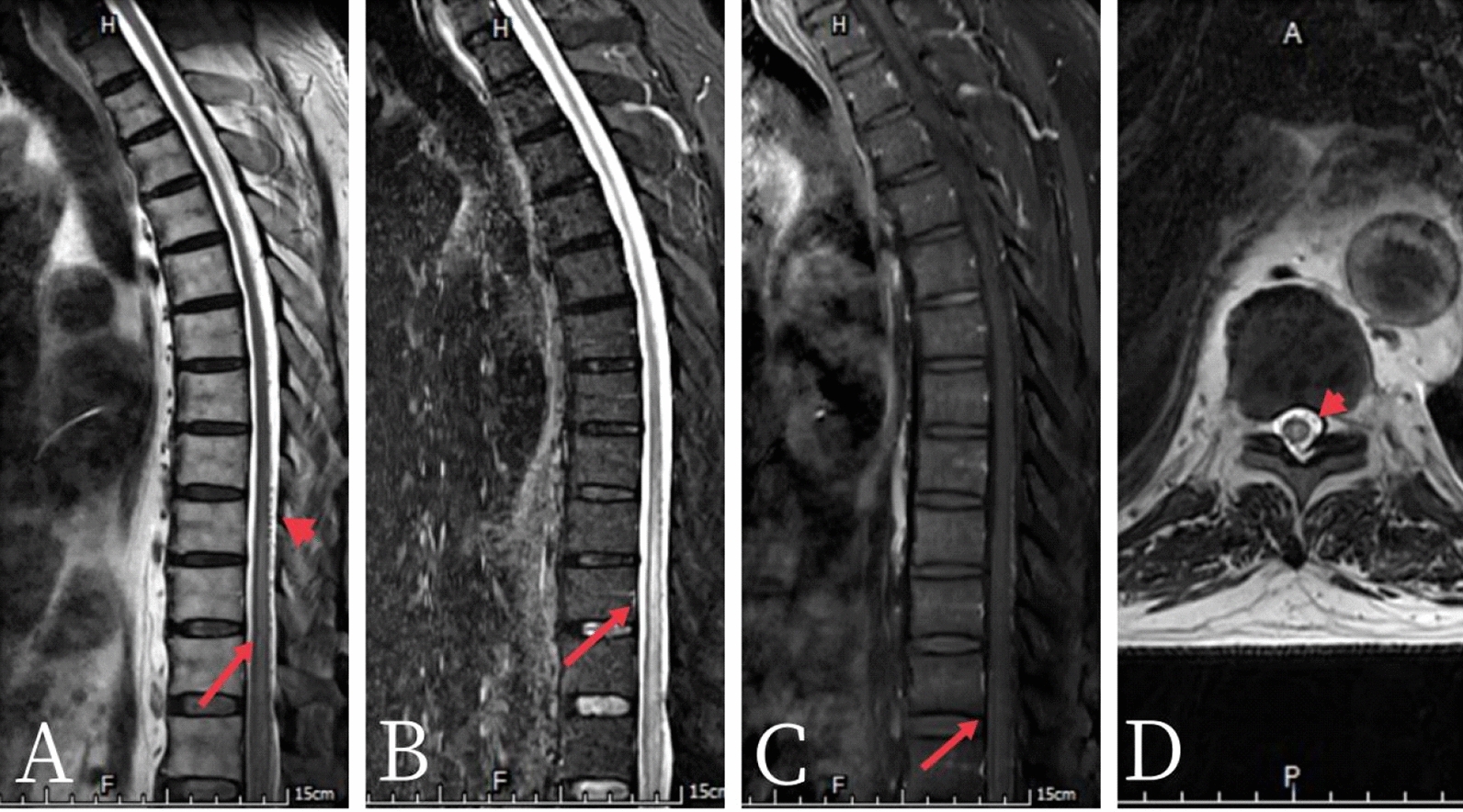
Enhanced MR images of the thoracic vertebrae: A-D show the T2, STIR, enhanced sequence and axial scans, respectively. Multiple strips of slightly longer T2 signal shadows were observed in the thoracic medulla at the level of the T8-12 vertebra, STIR showed high signal intensity, and mild linear enhancement was observed on the enhanced sequence (long red arrow). Multiple empty shadows of vascular flow were observed in the dorsal spinal cord of the corresponding segments (short red arrows)

**Fig. 2:**
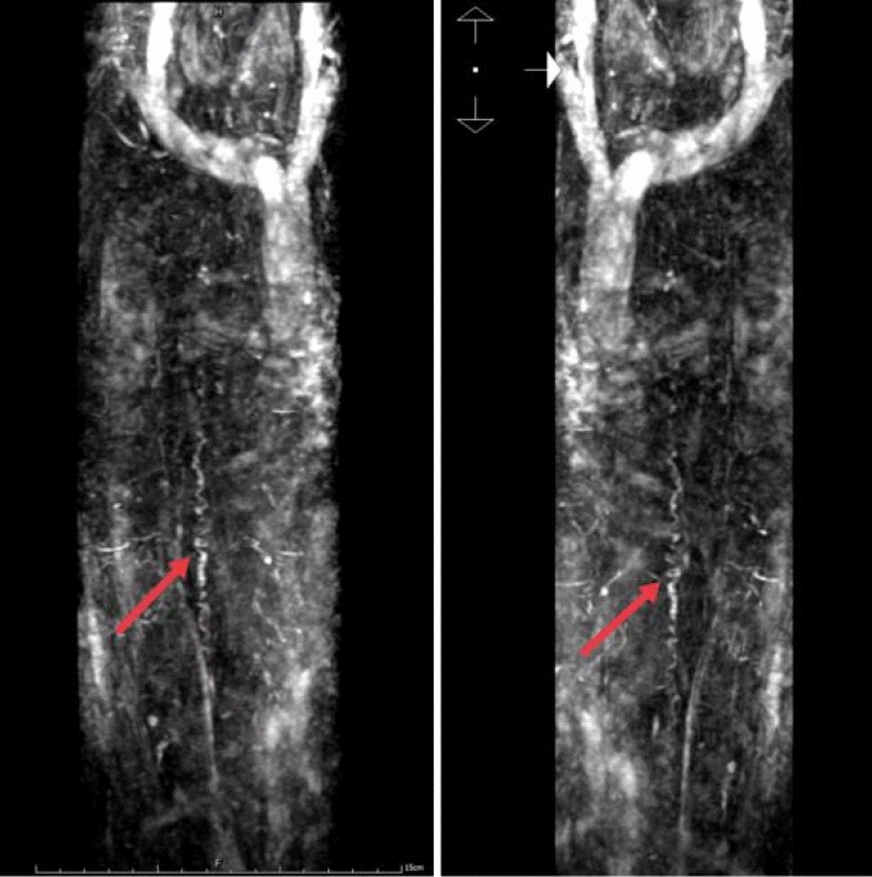
Contrast-enhanced magnetic angiography (CEMRA) of the spinal cord: Varicose veins were observed in the spinal nerve root veins of the corresponding segments

**Fig. 3:**
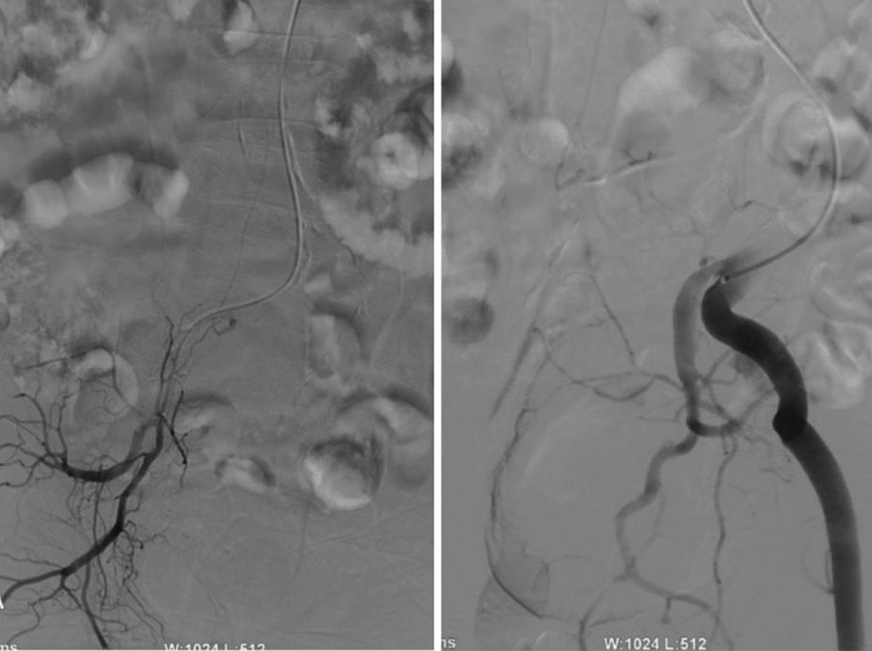
Angiography of the spinal vasculature and internal iliac artery: arteriovenous fistula staining was observed in the bilateral internal iliac arteries, and the fistulas were located in branches of the bilateral internal iliac artery. Both sides are drained to the level of the 6th intercostal artery through the tortuous thickened veins of the spinal cord

A total of 17 days later, the patient was referred to the neurology department of our hospital, and enhanced MRI of the thoracic vertebra revealed multiple abnormal signals in the thoracic medulla at the level of the T8–12 vertebra and a visible vascular shadow on the dorsal spinal cord (Fig 1). The possibility of spinal cord ischemia caused by arteriovenous malformations was then considered after completing contrast-enhanced magnetic angiography (CEMRA) of the spinal cord (Fig 2). Bilateral spinal dural arteriovenous fistulas (fistulas located in a branch of the bilateral internal iliac artery, with both sides draining to the level of the sixth intercostal artery through the tortuous thickened veins of the spinal cord) were confirmed after spinal vascular angiography 26 days later (Fig 3). Bilateral spinal dural arteriovenous fistula embolization was performed 33 days post-onset. Compared with complete paraplegia (0/5 muscle strength of both lower limbs) at baseline, there was a slight improvement in muscle strength in both distal lower extremities after embolization.

His physical examination at the time of admission revealed that his muscle strength was 5/5 in the upper extremities; 0/5 in the bilateral hip flexor muscle group; 2/5 in the bilateral knee extensor muscle group, ankle dorsiflexor muscle group, and ankle plantar flexor muscle group; 2/5 in the left toe extensor muscle group; and 0/5 in the right toe extensor muscle group on the basis of the Global Oxford Scale. Spasticity was not observed. Bilateral knee and left ankle reflexes were weakened, and the right ankle reflex was not elicited. Babinski reflex was positive in the left foot and not evoked in the right foot. On the basis of sensory examination, hypoesthesia was observed starting from the T12 level. No obvious anal contraction was observed in the digital diagnosis, but deep anal pressure and shallow anal sensation were retained, and the bulbocavernosus reflex was present. Rehabilitation evaluation results were as follows: the neurological level of injury was at T12, and the impairment scale (AIS) grade was B (incomplete injury) according to the American Spinal Injury Association (ASIA) 2019 classification, the zones of partial motor preservation were left S1 and right S1, respectively. Modified Barthel index was 20/100. Modified Aminoff-Logue Scale (mALS) score was 11/11, indicating severe disability (gait—requires a wheelchair “5,” urination—persistent incontinence or retention “3,” and defecation—persistent incontinence “3”). The results of the auxiliary examinations were as follows: ultrasonography of blood vessels in both lower limbs revealed no venous thrombosis. Urodynamic examination revealed a low-compliance bladder with decreased bladder volume in the urinary storage period, and abdominal pressure-assisted initiation of urination, detrusor hyperreflexia, and low-flow urination in the urination stage.

The rehabilitation treatment was as follows: limb motor function training, including basic movement training, muscle strength training, balance training, electric standing bed, neuromuscular electrical stimulation, and lower limb exoskeleton robot to improve lower limb motor function. Abdominal interference electricity to improve the neurogenic bladder (a muscarinic receptor antagonist was also given to relieve overactive bladder) and the neurogenic bowel. An air pressure-circulating pump was used to prevent thrombosis of the lower extremity vein. Each training session lasted 20 minutes, once per day, 5 days per week for 4 weeks.

**Fig. 4:**
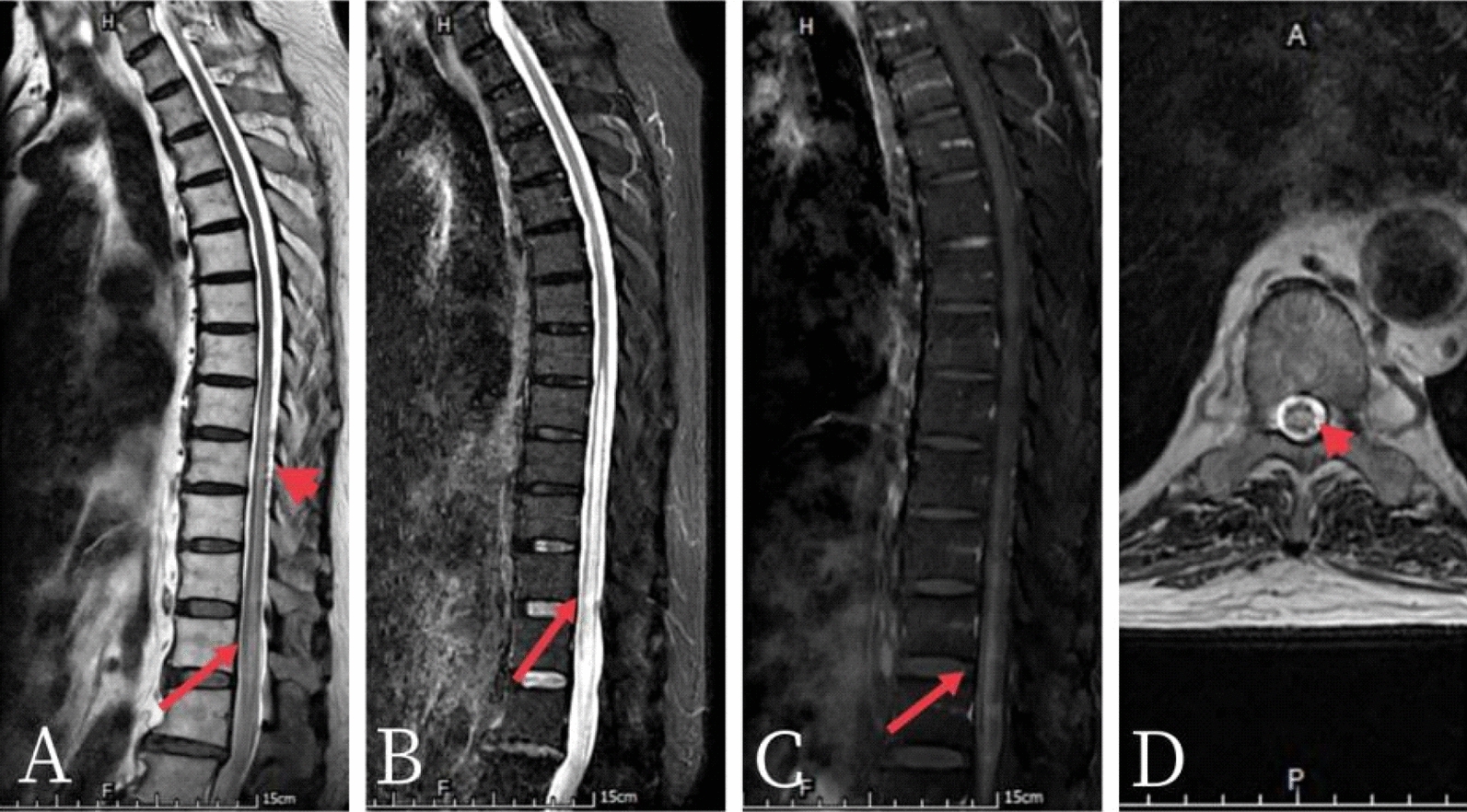
Enhanced MR image of the thoracic vertebra after 4 weeks of rehabilitation training A-D show the T2, STIR, enhanced sequence and axial scans, respectively. The multiple abnormal signals and linear enhancement signals (long red arrow) in the thoracic spinal cord were similar to those in the previous examination. The empty shadows of vascular flow (short red arrows) were slightly decreased compared to before

**Fig. 5:**
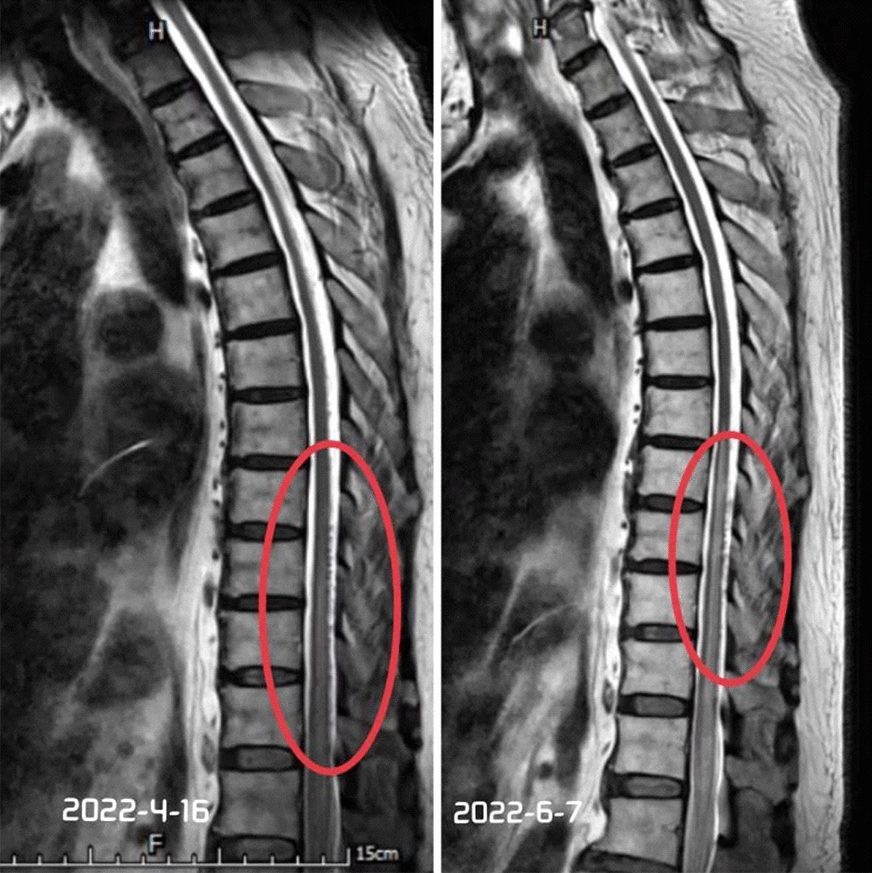
Comparison of MR images of the thoracic vertebrae before and after treatment. There were fewer vascular empty shadows on the dorsal spinal cord than before treatment

After 4 weeks of rehabilitation training, the re-examined enhanced MRI of the spinal cord showed that the vascular empty shadows on the dorsal spinal cord had decreased than before treatment (Fig 4, Fig 5), the motor function of both lower limbs slightly improved. The results of the physical examination are compared with those at admission in Table 1. Rehabilitation evaluation revealed that the neurological injury level was at L1, the AIS grade was B (incomplete injury) according to the ASIA classification, and the zones of partial motor preservation were left S1 and right S1, respectively. Modified Barthel index was 33/100, mALS score was 11/11, and 6 months after disease onset, patient underwent clean intermittent catheterization (CIC) and still had difficulty defecating. Patient’s lower limb weakness improved somewhat, but he could still not stand or walk, and 1 year later, patient could roll over independently but was still unable to stand or walk.

**Table 1:** Physical examination at admission and 4 weeks after rehabilitation

	Muscle strength of lower extremities						Tendon reflexes		Babinski reflex	Level of hypoesthesia	Voluntary anal contraction	Deep anal pressure	Bulbocavernosus reflex
		Hip flexor	Knee extensor	Ankle dorsiflexor	Toe extensor	Ankle plantar flexor	Knee reflex	Ankle reflex					
On admission	Left	0	2	2	2	2	(+)	(+)	(+)	T12	No	Yes	Present
	Right	0	2	2	0	2	(+)	(−)	(−)				
After 4 weeks	Left	2+	4	2+	2	2	(+)	(+)	(+)	L1	No	Yes	Present
	Right	2+	4	2	1+	2	(++)	(+)	(−)				

## Discussion

In 1974, Aminoff first proposed that the pathophysiological mechanism of SDAVF is the direct anastomosis of nerve root arteries and veins to form an orifice fistula, which leads to intraspinal venous hypertension, spinal cord congestion, and edema. Subsequently, ischemic necrosis develops, which results in severe neurological impairment [8].

SDAVF mostly has an insidious onset, with progressive aggravation and long disease course, but 5–15% of patients experience acute myelopathic exacerbation [9]. However, all of these reported cases of acute aggravation have a clear trigger, which includes corticosteroid use, lumbar puncture, long periods of standing, prolonged prone positioning during surgery or other treatments, singing, and Valsalva maneuvers [9–16] (Table 2). When SDAVF is acutely exacerbated, venous pressure increases, resulting in a decrease in blood supply from the spinal arteries to the veins, which then causes vascular congestion, spinal cord edema, and ischemia [17]. Nevertheless, in this case, the patient experienced an acute onset of SDAVF and rapid progression to paraplegia, which peaked in severity within 4 days of onset. Moreover, the patient developed the disease during sleep without an obvious cause. No cases of acute-onset SDAVF without an obvious trigger have been reported. A possible hypothesis for the cause of this SDAVF is that the patient experienced a pressure gradient change caused by an increased intrathecal pressure as a result of lying in a prone position during sleep.

**Table 2:** Characteristics of the published case reports of SDAVF with acute aggravation

Study	Sex	Age (years)	Time of diagnosis (months)	Function at its worst	Triggers for aggravation	SDAVF location	Intervention	Prognosis
Park *et al*. (2021)	Male	49	2	Complete paraplegia with dysesthesia and urinary and fecal incontinence	Electroacupuncture on his back	T12–L1	Surgery	Motor function markedly improved at immediate postoperative period, able to walk on his own at postoperative 1 month, able to run and climb up the staircase without difficulty at postoperative 1 year
Vini *et al*. (2002)	Male	72	36	Need to seat while singing to avoid collapsing, able to walk independently	Singing	L1	Surgery	Able to sing while standing throughout almost his entire performance, and his lower limb strength was continuing to improve at 3 months after surgery
Kim *et al*. (2016)	Male	54	12	Complete paraplegia with dysesthesia and urinary and fecal incontinence	Transforaminal epidural steroid injection	T6	Endovascular embolization	46 days after intervention, the strength of both lower extremities was improved to 4/5–5/5, gait disturbance and failure to void persisted at 1-year follow-up
Kim *et al*. (2016)	Female	64	4	Complete paraplegia with dysesthesia and urinary and fecal incontinence	Epidural steroid injection	Sacral branch of the left internal iliac artery	Endovascular embolization	10 days later, the motor strength of lower extremities was 2/5–3/5 in the right leg and 4/5–5/5 in the left leg. Spastic paraplegic features persisted when she was followed up after 3.5 years
DiSano *et al*. (2017)	Female	63	26	Bilateral lower extremity paraparesis with dysesthesia	Intravenous corticosteroid administration	L2	Surgery	At 2/month follow-up, patient had some mild subjective improvement in strength and some sensory improvement
García-Cabo *et al*. (2017)	Male	68	12	Spastic paraplegia and hypesthesia	Lumbar puncture	Dural branches of a radicular artery	Surgery	During the following months, paraplegia slightly improved
Arman Çakar *et al*. (2018)	Male	41	0.25	Complete paraplegia	LUMBAR puncture	T6–7	Surgery	Patient’s paraplegia recovered to paraparesis with MRC (Muscle Strength Grading) grade 3/5 after surgery. After 2 months his paresis recovered entirely
Noh *et al*. (2017)	Female	72	–	Bilateral deltoid and lower extremity weakness with diffuse hyperreflexia	High-volume lumbar puncture	C5–6	Endovascular embolization	1 month later, she was back to her original neurological baseline
Thiru *et al*. (2017)	Male	48	8	Bilateral lower extremity paraparesis with dysesthesia and urinary and fecal incontinence	Lumbar epidural steroid injection	Left lateral sacral artery	Endovascular embolization	2 weeks after embolization, he made significant improvement in functional independence and a near-complete recovery of muscle strength

Clinical manifestations of SDAVF mainly include varying degrees of motor, sensory, urination, and defecation dysfunction. Due to its low incidence and likelihood of being overlooked on vascular images, as well as the low specificity of clinical examinations, delayed diagnosis and misdiagnosis are highly probable. The median time between symptom occurrence and diagnosis is 10.15 months [5, 18], and the maximum time is 240 months [19]. Patients can be commonly misdiagnosed with degenerative disc disease, demyelination, myelitis, intramedullary tumors, spinal cord infarction, spinal arteriovenous malformation (SAVM), Guillain–Barré syndrome, or other conditions [18, 20, 21]. This patient was initially misdiagnosed with inflammatory demyelinating myelopathy, as previously reported.

The most commonly reported cause of acute exacerbation of SDAVF was corticosteroids use after misdiagnosis. A prospective cohort study [22] confirmed this finding, and the aggravated condition may persist despite successful obliteration of the fistula. This patient had no history of steroid hormone drug use or other possible triggers before the onset of the disease, however, low-dose steroids were used for 5 days during the course of the disease due to misdiagnosis, which did not cause a change in the condition because of the peak severity of the condition at the time of presentation, but it may be associated with the poor prognosis.

SDAVFs develop more frequently in men than in women, particularly those who are older than 40 years [5]. Fistulas are usually located in the thoracic spine. Babichev *et al*. [5] reviewed previous studies and analyzed the clinical data of 187 patients, which revealed that the thoracic spine is the most frequent location of SDAVFs, mainly at the T6, T7, and T9 vertebral levels. Furthermore, motor disorders are more severe when the fistula is located at or below the T9 vertebra. In this patient, the lesion was located at the T8–12 vertebral level, which is consistent with the most common site of SDAVF development, and lesions at this level are often accompanied by more severe symptoms.

Prognostic factors for SDAVF include age, severity, early diagnosis, and treatment. The degree of neurological dysfunction is the main factor determining the prognosis of patients[1, 20, 21]. A prospective cohort study conducted by Yongjie Ma [1] evaluated the 1-year prognosis of 94 patients with cervical and thoracolumbar SDAVFs to identify short-term clinical outcomes and prognostic factors. The results showed that approximately four-fifths of patients experienced clinical improvement at 12 months and confirmed that the preoperative mALS score was the strongest predictor of clinical improvement in the cohort. Early rehabilitation training after surgical treatment may have a positive impact on prognosis [23]. However, this patient experienced acute onset, rapid progression, severe preoperative neurological dysfunction, and had a mALS score of 11; endovascular embolization was performed 2 weeks later, and rehabilitation training was started 40 days later, but only minimal improvement was observed after 1 year. Therefore, although endovascular treatment and rehabilitation training were performed promptly after early diagnosis, the patient experienced only minimal and slow clinical improvement. Further research is necessary to confirm this result, and determine whether there would be different outcomes if microsurgery was performed.

## Conclusion

This case serves as a warning to the fact that a diagnosis of SDAVF cannot be excluded in acute-onset, rapidly progressing spinal cord lesions, and clinicians need to be knowledgeable of the specific clinical manifestations and MRI features of SDAVF to make an early diagnosis. To avoid exacerbation of the condition due to inappropriate treatment, spinal vascular examination is necessary to identify vascular malformations. Moreover, patients with SDAVF with acute-onset, rapid progression, and severe neurological dysfunction may have poor prognosis even after prompt surgical treatment.

## Data Availability

Data sharing is not applicable to this article as no datasets were generated or analyzed during the current study.
